# TNFa/TNFR2 signaling is required for glial ensheathment at the dorsal root entry zone

**DOI:** 10.1371/journal.pgen.1006712

**Published:** 2017-04-05

**Authors:** Cody J. Smith, Michael A. Wheeler, Lindsay Marjoram, Michel Bagnat, Christopher D. Deppmann, Sarah Kucenas

**Affiliations:** 1 Department of Biology, University of Virginia, Charlottesville, Virginia, United States of America; 2 Neuroscience Graduate Program, University of Virginia, Charlottesville, Virginia, United States of America; 3 Department of Cell Biology, Duke University, Durham, North Carolina, United States of America; Fred Hutchinson Cancer Research Center, UNITED STATES

## Abstract

Somatosensory information from the periphery is routed to the spinal cord through centrally-projecting sensory axons that cross into the central nervous system (CNS) via the dorsal root entry zone (DREZ). The glial cells that ensheath these axons ensure rapid propagation of this information. Despite the importance of this glial-axon arrangement, how this afferent nerve is assembled during development is unknown. Using *in vivo*, time-lapse imaging we show that as centrally-projecting pioneer axons from dorsal root ganglia (DRG) enter the spinal cord, they initiate expression of the cytokine TNFalpha. This induction coincides with ensheathment of these axons by associated glia via a TNF receptor 2 (TNFR2)-mediated process. This work identifies a signaling cascade that mediates peripheral glial-axon interactions and it functions to ensure that DRG afferent projections are ensheathed after pioneer axons complete their navigation, which promotes efficient somatosensory neural function.

## Author summary

To survive, animals must receive sensory information from their environment and relay it to the central nervous system. The efficient transfer of this environmental information to the brain and spinal cord requires both neuronal and glial populations found along peripheral sensory nerves. In this work, we describe the early steps of sensory nerve assembly. We identify that a molecule most well described in inflammation, tumor necrosis factor alpha (TNFa), and its obligate receptor are utilized to control glial ensheathment of sensory axons. This ensheathment occurs shortly after the first sensory axons enter the spinal cord. Together, our work uses time-lapse imaging and genetic manipulation to demonstrate the orchestrated coordination of glial and neuronal cells during nerve formation, providing insight into how sensory information from the periphery is integrated into the CNS during development.

## Introduction

Efficient somatosensory perception requires that neurons propagate sensory stimuli from the periphery to the central nervous system (CNS). Cutaneous somatosensory stimuli are first detected by excitable neurites in the skin, transduced past the dorsal root ganglion (DRG) cell soma and then conveyed to the CNS via a centrally-projecting afferent axon [1–3]. Glial cells that ensheath these components ensure that this somatosensory information is efficiently and rapidly propagated [4–6].

During development, the sensory neurons and glia that ensheath them are specified from neural crest cells that migrate from their dorsal neuroepithelial origin [7,8]. These neural crest cells eventually coalesce to form DRG, which continue to add neurons throughout embryonic development [7,8]. Each sensory neuron then produces a pseudobipolar axon that innervates its peripheral target and a central projection that crosses into the spinal cord at the dorsal root entry zone (DREZ) [1–3]. In mice at late embryonic stages, glial cells located at the DREZ are thought to act as positive substrates for axonal entry into the spinal cord [9]. However, our understanding of how DRG pioneer axons enter the spinal cord and how they influence development of associated glia is unknown.

In addition, although our understanding of molecular pathways that mediate DRG neuronal differentiation and specification are abundant [10], we know very little about the molecular pathways that are required for the development of sensory glia within the DRG [11]. In fact, although there is a growing body of literature of known signaling pathways that mediate glial association and ensheathment of axons in the periphery [11–17], it is still limited compared to our understanding of neuronal development [5,18]. The most well characterized molecular mechanism that controls peripheral axon-glial communication is the Neuregulin 1 Type III (Nrg1-III) ligand and the ErbB family of receptors [5,19,20]. Interestingly, however, genetic knockouts that disrupt Nrg1-III/ErbB signaling have peripheral axons with associated glia, suggesting that additional mechanisms likely function to ensure glia ensheath peripheral axons [21,22].

To identify signaling pathways that mediate glial ensheathment of DRG pioneer axons, we took a multipronged approach, merging the elegance of zebrafish *in vivo*, time-lapse imaging with the power of mouse genetics. Live imaging in zebrafish revealed that pioneer DRG axons navigate to the DREZ with glial cells closely associated with the growth cone. We demonstrate that the cytokine tumor necrosis factor alpha (TNFa) is upregulated in pioneer axons upon spinal cord entry and that a TNFa/TNF receptor 2 (TNFR2) pathway directs ensheathment of the central projection, as genetic disruption of either TNFa or TNFR2 lead to a failure of glial ensheathment of pioneer axons in both mice and zebrafish. Together, these data identify critical cellular determinants of sensory, central projection formation and a mechanism underlying peripheral axon-glial communication.

## Results

### Pioneer DRG axons navigate directly to the DREZ

**Fig 1:**
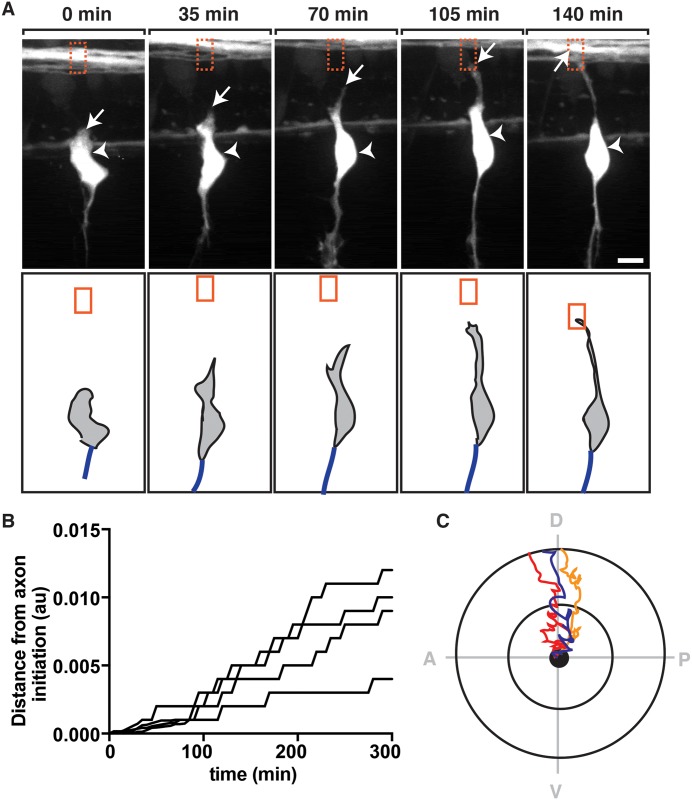
DRG pioneer axons navigate directly to the DREZ. (A) Images and traced schematics from a 24 hour time-lapse movie starting at 48 hpf in a *Tg(ngn1*:*egfp)* embryo showing navigation of the DRG pioneer axon toward the DREZ. Orange box indicates location of DREZ. Arrows denote the growth cone and arrowheads denote location of cell body. Blue process in schematic denotes peripheral axon. (B) Quantification of the distance (y-axis) and time (x-axis) of 4 growth cones as they navigated toward the DREZ. (C) Schematic tracings of 3 pioneer axons as they navigated to the DREZ. Compass shows Dorsal-D, Ventral-V, Anterior-A, Posterior-P. Scale bar, 25 μm.

Sensory afferent nerves are ensheathed by peripheral glia from the DRG to the edge of the spinal cord. However, the precise spatiotemporal dynamics of this cellular arrangement at the DREZ are not understood. Therefore, we characterized early stages of DREZ development to understand how the primary afferent circuit is established. We started by investigating how DRG afferent axons enter the spinal cord. To achieve this, we directly visualized the navigation of pioneer axons to the DREZ. Using *Tg(ngn1*:*egfp)* zebrafish, which have regulatory sequences of *neurogenin1* (*ngn1*) driving expression of GFP in developing DRG neurons [23], we began imaging embryos at 48 hours post fertilization (hpf), prior to central and peripheral axon formation (Fig 1A, S1 Movie). Beginning at this stage, newly specified DRG neurons had transient, filopodia-like structures that projected ventrally initially, followed by similar projections that extended dorsally toward the presumptive DREZ (Fig 1A, S1 Movie). These filopodia-like structures transitioned into single axons, and between 48 and 56 hpf, the dorsal processes transitioned into a growth cone that extended dorsally away from the cell soma (Fig 1A, S1 Movie). The growth cone eventually ceased its dorsal migration and then entered the spinal cord at a position that corresponded with the location of the dorsolateral fasciculus (DLF) between 50 and 72 hpf (Fig 1A, S1 Movie). By 72 hpf, each somite along the anterior-posterior (AP) axis of the larva contained a centrally-projecting DRG axon that had entered the DREZ.

To characterize the velocity and trajectory of these pioneer axons as they migrated toward the DREZ, we traced growth cones in four individual movies similar to S1 Movie. Tracing the most dorsal location of the growth cone at each time point revealed that DRG pioneer axons navigated with constant velocity toward the DREZ (Fig 1B, S1 Movie). When this tracing was plotted to represent the path of the growth cone, we saw that axons navigated directly dorsally from the cell body to the DLF (Fig 1C, S1 Movie). Based on these data, we conclude that the DRG pioneer axon has a striking ability to navigate directly to the DREZ.

### Glia navigate with pioneer axons to the DREZ

**Fig 2:**
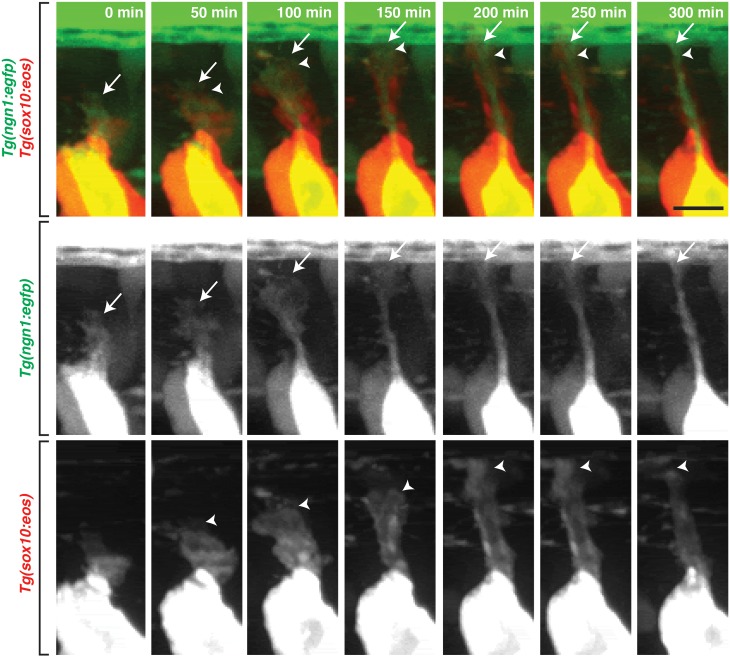
DRG glia navigate with pioneer axons. Images from a 24 hour time-lapse movie starting at 48 hpf in a *Tg(sox10*:*eos);Tg(ngn1*:*egfp)* embryo that was exposed to UV light to photoconvert Eos in the whole embryo. These frames show the pioneer axon (green) and associated glia (red) migrate together to the DREZ. Below are individual channels for GFP and Eos. Arrows denote the pioneer axon growth cone and arrowheads denote location of the DRG glia. Scale bar, 25 μm.

We next sought to determine when glial cells associated with nascent sensory afferents during navigation to the DREZ. To do this, we used *Tg(sox10*:*eos);Tg(ngn1*:*egfp)* embryos, which label DRG precursors with Eos, and DRG sensory neurons with GFP [23,24]. We imaged these animals from 48 to 72 hpf to capture glial dynamics in relation to the DRG pioneer axon. Early in development, sensory neurons are derived from *sox10*^+^ neural crest cells and begin expressing *ngn1* when they become post-mitotic neurons [24]. Therefore, in our imaging, sensory neurons were *ngn1*^+^ (green) and also transiently expressed Eos (red) and appeared yellow. All other non-neuronal cells or glia were marked with Eos only and appeared red (Fig 2, S2 Movie). Time-lapse imaging of these embryos, beginning at 48 hpf, revealed that as the pioneer growth cone navigated dorsally toward the DREZ, *sox10*^+^ glial cells lagged slightly behind the growth cone (Fig 2, S2 Movie). Once the pioneer axon reached the level of the DLF, the growth cone entered the spinal cord while associated *sox10*^+^ glia remained in the periphery, associated with the central afferent projection from the DRG to the edge of the spinal cord at the DREZ (Fig 2, S2 Movie). In these images, the axon and associated glial cell can be distinguished based on the distribution of fluorescent protein which shows an axon with a concentrated fluorescent projection and a glial cell with fan-like morphology and more diffuse distribution of fluorescent protein. Based on these data, we conclude that glial cells navigate with the pioneer growth cone to the DREZ before they ensheath the peripheral arm of the afferent axon.

### TNFa signaling is essential for glial ensheathment at the DREZ

**Fig 3:**
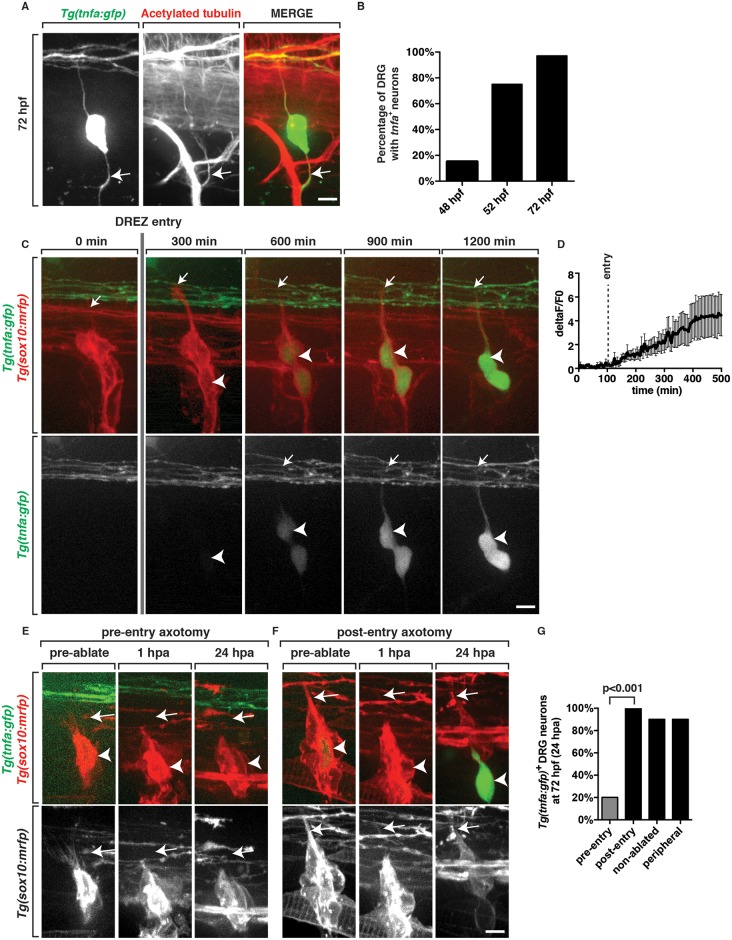
DRG neurons upregulate TNFa after pioneer axons enter the spinal cord. (A) At 72 hpf, *Tg(tnfa*:*gfp)* zebrafish embryos show robust expression of GFP in DRG neurons after the pioneer axon has entered the spinal cord. Arrow demarcates the peripheral projection labeled with acetylated tubulin. (B) Quantification of *tnfa*^+^ DRG neurons at 48, 52 and 72 hpf (n = 30 DRG nerves). (C) Excerpt from a 24 hour time-lapse movie starting at 48 hpf in a *Tg(tnfa*:*gfp);Tg(sox10*:*mrfp)* embryo shows GFP expression in DRG neurons increasing as the pioneer axon (arrow), identified by RFP, enters the spinal cord. Arrowhead denotes DRG neuron cell soma. (D) Intensity profile of GFP in movie shown in panel C (n = 7 DRG). (E) Live images of a 48 hpf *Tg(tnfa*:*gfp);Tg(sox10*:*mrfp)* embryo showing a pioneer axon (arrow) just as it has formed pre-axotomy, 1 hpa and 24 hpa. In these images, axotomy prevents pioneer axons from entering the spinal cord and GFP expression is never observed. Arrowhead denotes DRG neuron cell soma. (F) Live images of a 48 hpf *Tg(tnfa*:*gfp);Tg(sox10*:*mrfp)* embryo where the pioneer axon (arrow) had already entered the spinal cord. In this instance, axotomy did not affect GFP expression. (G) Quantification of GFP expression in DRG neurons from panels E and F (n = 5 nerves). Scale bars, 25 μm.

We next sought to identify the molecular mechanism that mediates glial ensheathment of DRG pioneer axons. In our search for transgenic lines that labeled DRG neurons, we identified a new transgenic line, *Tg(tnfa*:*gfp)*, which uses *tumor necrosis factor α* (*tnfa*) regulatory sequences to drive expression of GFP in zebrafish DRG neurons (Fig 3A) [25]. This expression pattern is consistent with the expression of TNFa in mouse DRG neurons during development [26]. To confirm that *Tg(tnfa*:*gfp)* is a faithful reporter of endogenous *tnfa* mRNA expression in DRG neurons, we used multiplex fluorescent *in situ* hybridization (FISH) probes, which tile the *tnfa* transcript with 37, 20-nucleotide RNA probes directly conjugated to a fluorescent reporter [27]. We performed this analysis in *Tg(ngn1*:*gfp)* larvae at 72 hpf and visualized that *tnfa* expression co-localized with GFP^+^ sensory neurons (S1A Fig), consistent with the hypothesis that *Tg(tnfa*:*gfp)* recapitulates endogenous *tnfa* expression.

Interestingly, we noticed that in *Tg(tnfa*:*gfp)* embryos, GFP expression was only detected in DRG neurons at 72 hpf, but not at 48 hpf (Fig 3A and 3B, 48 hpf-18%, 72 hpf-97%, n = 30 DRG). When *Tg(tnfa*:*gfp)* expression was scored at 52 hpf, we observed GFP^+^ neurons predominantly in anterior DRG that were first to send pioneer axons into the spinal cord and an absence of GFP expression in posterior DRG that had yet to send pioneer axons to the DREZ (Fig 3B, 52 hpf-75%, n = 30 DRG). We therefore hypothesized that *tnfa* may be selectively expressed only in DRG neurons that had formed a pioneer axon that had entered the CNS. To test this hypothesis, we used time-lapse imaging between 48 and 72 hpf in *Tg(tnfa*:*gfp);Tg(sox10*:*mrfp)* embryos, when pioneer DRG axons navigate into the spinal cord. Using this double transgenic line, we were able to visualize entry of the growth cone with the *Tg(sox10*:*mrfp)* line because DRG neurons transiently express *sox10* before upregulating *tnfa* expression. When we quantified the intensity of *Tg(tnfa*:*gfp)* from the movie represented in Fig 3C, we noted that GFP was not detectable in DRG neurons before the pioneer axon entered the spinal cord, but increased after entry (Fig 3C and 3D, S3 Movie). By 72 hpf, GFP intensity was brightly expressed in nearly all DRG neurons along the trunk of the zebrafish and this profile was consistently visualized (Fig 3B and 3C, S3 Movie). Based on these data, we demonstrate that *tnfa* is upregulated in DRG neurons as pioneer axons enter the spinal cord.

To test if *Tg(tnfa*:*gfp)* expression in DRG neurons required pioneer axon navigation into the spinal cord, we performed axon transections using a pulsed nitrogen dye laser to perturb pioneer axon entry into the CNS [28]. Using *Tg(tnfa*:*gfp);Tg(sox10*:*mrfp)* embryos, we visualized and axotomized DRG pioneer axons that could be identified as RFP^+^ and GFP^−^ at 48 hpf. We scored successful axotomy by an aberration of the filopodia-like projections at the growth cone of axotomized axons (S2 Fig). In these ablations we targeted the growth cone and did not see any debris that corresponded with RFP^+^ glia. At 72 hpf, 24 hours post axotomy (hpa), we detected a reduction in GFP^+^ DRG neurons (20% GFP^+^ n = 5 DRG) (Fig 3E and 3G). To confirm that this lack of expression was not a response to axotomy, we axotomized GFP^+^ axons that had already entered the spinal cord at 48 hpf. 24 hpa, we did not visualize an elimination or reduction of GFP expression in these DRG neurons (Fig 3F and 3G). These data demonstrate that axons that have entered the spinal cord continue to express *Tg(tnfa*:*gfp)* even after axotomy. However, we acknowledge that post-transcriptional delay in protein detection may mask underlying changes in gene expression. We therefore also axotomized the peripheral projection of the DRG neuron before the afferent axon had projected into the spinal cord and scored GFP expression at 72 hpf. Similarly, peripheral axotomy also did not reduce the number of GFP^+^ neurons at 72 hpf (Fig 3G). Taken together, these data are consistent with the hypothesis that spinal cord entry is required for *Tg(tnfa*:*gfp*) expression in DRG pioneer axons.

**Fig 4:**
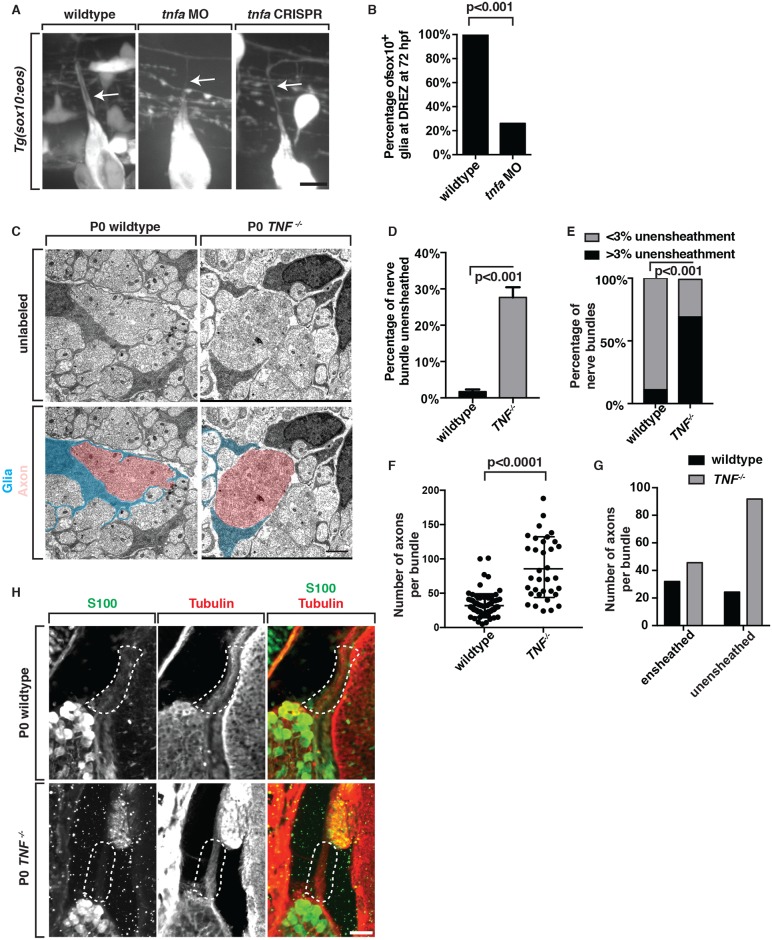
TNFa is required for axonal ensheathment by DREZ glia. (A) Images of wildtype, *tnfa* morphant and *tnfa* F0 CRISPR mutant *Tg(sox10*:*eos)* larvae at 72 hpf. Arrow denotes central branch that is ensheathed by *sox10*^+^ glia in wildtype but not *tnfa* morphants or *tnfa* F0 CRISPR mutants. (B) Quantification of data from panel A, wt n = 40 DRG, *tnfa* MO n = 54 DRG. (C) Electron microscopy images of DRG tissue from wildtype and *TNF*^*-/-*^ P0 pups showed a reduction in the ensheathment of axonal bundles. (n = 3 animals/genotype). (D,E) Quantification of ensheathed vs unensheathed bundles in wildtype and *TNF*^*-/-*^ pups (3 animals, wt n = 108 nerve bundles, *TNFR2-/-* n = 85 nerve bundles). (F,G) Quantification of the number of axons per small-caliber bundle in wildtype and *TNF*^*-/-*^ pups. (H) Images of spinal column tissue from wildtype and *TNF*^*-/-*^ P0 embryo labeled with antibodies to S100 (green) and βIII Tubulin (red). Traced area denotes central projection that extends into the spinal cord. In *TNF*^*-/-*^ pups, the central afferent lacks S100 staining. Fisher’s exact test (B,E,G). Students t-test (D,F). Scale bars, 25 μm (A,H), 5 μm (C).

Given the upregulation of TNFa after the pioneer axon enters the spinal cord, we hypothesized that TNFa-mediated signaling would be important in the maintenance of pioneer axons and/or the glia that ensheath them. To investigate this hypothesis, we injected a *tnfa* translation blocking morpholino oligonucleotide (MO) [29] into single-cell *Tg(sox10*:*eos)* embryos and characterized the outcome of perturbed TNFa signaling on DRG pioneer axon-associated glia. In *Tg(sox10*:*eos) tnfa* morphants at 72 hpf, we visualized *sox10*^+^ axonal projections, but did not detect any *sox10*^+^ glia ensheathing pioneer axons (26% contained *sox10*^+^ glia, n = 54 DRG), which is in contrast to the full ensheathment we observed in wildtype control larvae (100% contained *sox10*^+^ glia, n = 40 DRG) (Fig 4A and 4B). We extended our analysis of these experiments to determine if this phenotype was a delay in glial ensheathment by scoring the ensheathment phenotype at 96 hpf. Similarly, at 96 hpf, we visualized defective ensheathment in *tnfa* morphants when compared to wildtype larvae (36% contained *sox10*^+^ glia, n = 61 DRG).

To confirm these MO-injected phenotypes were specific to perturbation of *tnfa*, we utilized the CRISPR/cas9 system to generate F0 *tnfa* mutants [30,31] (S2 Table). To do this, we injected *Tg(sox10*:*eos)* embryos with guide RNAs (gRNA) specific to *tnfa* and scored glial ensheathment of DRG pioneer axons at the DREZ at 72 hpf. Consistent with our MO data, glial ensheathment was disrupted in F0 *tnfa* gRNA-injected larvae (wildtype– 99% contained *sox10*^+^ glia, n = 160 DRG, *tnfa–* 67% contained *sox10*^+^ glia, n = 160 DRG, p<0.0001 students t-test) (Fig 4A, S3C Fig). To confirm that these injections induced mutations that disrupted the genomic region of *tnfa*, we performed a T7 endonuclease assay (S3A Fig) [30,31] and detected that 7 out of the 13 selected larvae that were assayed showed potential mutations. We then confirmed this analysis by sequencing *tnfa* in the specific larvae that showed a potential mutation in the T7 assay and had a glial ensheathment phenotype (S3D Fig) [30,31].

We next asked if this role for TNFa in glial ensheathment was evolutionarily conserved in mice. To test this hypothesis, we measured ensheathment of the DRG afferent nerve in wildtype and *Tnfsf1a*^-/-^ (*TNF*^*-/-*^) pups at P0 using electron microscopy. We first quantified this by calculating the percentage of an axon bundle that was ensheathed by glial membrane. In wildtype pups at P0, on average, 1.7% of individual axonal bundles were unensheathed (Fig 4C and 4D). Of these wildtype axonal bundles, only 11 nerve bundles of the 110 that we measured contained axons that were more than 3% unensheathed, indicating nearly every axon of the afferent nerve is ensheathed by birth (Fig 4C and 4E, n = 3 animals). In contrast, in *TNF*^*-/-*^ pups, we observed that 27.6% of individual axon bundles within the DRG afferent sensory nerve were unensheathed (Fig 4C–4E, n = 3 animals). We also observed several small caliber axons that lacked glial membrane around the entirety of the bundle mixed with large caliber axons that were also incompletely ensheathed, which caused mixing between these axonal bundles (Fig 4C). We therefore also quantified the number of axons in each small caliber bundle. In wildtype pups, small caliber bundles had on average, 32.0 axons, whereas *TNF*^*-/-*^ pups contained 84.8 axons (Fig 4F, n = 3 animals). When binning the axons bundles into ones that were ensheathed versus unensheathed, *TNF*^*-/-*^ pups contained 45.6 that were ensheathed compared to 91.8 that were unensheathed (Fig 4G, n = 3 animals). This is compared to wildtype that had 32.0 ensheathed and 24.3 unensheathed (Fig 4G, n = 3 animals). Based on these data and our analysis from zebrafish, we propose that *tnfa* is required for glial ensheathment of DRG pioneer axons.

To further test this model, we collected trunk tissue from P0 wildtype and *TNF*^*-/-*^ pups at P0, sectioned through the L3-L6 spinal column and stained tissue with the glial marker S100 and neuronal marker βIII Tubulin [26,32]. In wildtype pups, we observed S100^+^ cells associated with DRG Tubulin^+^ axons (Fig 4H). In contrast, we observed significantly reduced S100^+^ labeling along the DRG central projection in *TNF*^*-/-*^ pups (Fig 4H). To confirm this phenotype, we labeled *TNF*^*-/-*^ and wildtype tissue with additional glial markers, including ErbB3, a marker for glial precursors/immature glia in the PNS and Isl1, which is expressed in sensory neurons within the DRG [4,5]. Consistent with the hypothesis that glial ensheathment was disrupted in *TNF*^*-/-*^ pups, ErbB3 was reduced along Isl^+^ sensory afferent axons compared to wildtype pups (S4 Fig).

### TNFa activates NFkB in DRG pioneer axon-associated glia

**Fig 5:**
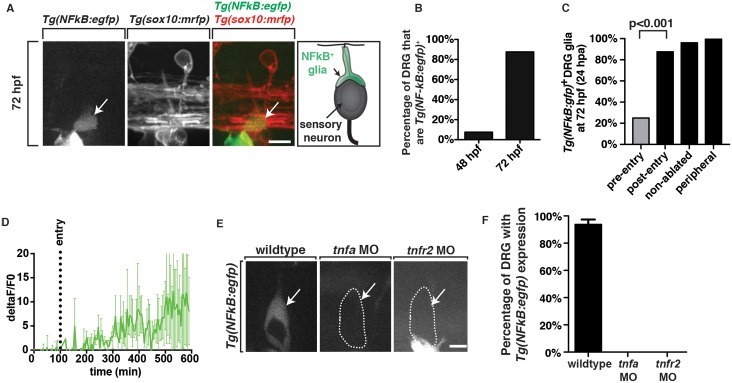
TNFa/TNFR2-mediated signaling is active in pioneer axon-associated glia. (A) Images and schematic of *Tg(NFkB*:*egfp);Tg(sox10*:*mrfp)* embryos at 72 hpf showing GFP^+^ glia at the DREZ. (B) Quantification of the percentage of DRG with *Tg(NFkB*:*egfp)* expression at 48 and 72 hpf, n = 30 DRG. (C) Quantification of NFkB^+^ DREZ glia along pioneer axons that were axotomized before entry (pre-entry), after entry (post-entry), non-ablated and peripheral axotomy (n = 8 nerves/condition). (D) Intensity profiles of GFP in *Tg(NFkB*:*egfp);Tg(sox10*:*mrfp)* embryos from 48 to 72 hpf showing GFP upregulation upon axon entry into the spinal cord. Shown is the average of 2 movies normalized for the time of entry. (E) Images of *Tg(NFkB*:*egfp)* embryos in wildtype, *tnfa* morphant and *tnfr2* morphant larvae showing NFkB activation is dependent on *tnfa* and *tnfr2*. (F) Quantification of data from panel D, SEM is shown, wt n = 30 DRG, *tnfa* MO n = 30 DRG, *tnfr2* MO n = 30 DRG. Scale bars, 25 μm.

To test the possibility that pioneer axon TNFa was signaling directly to associated glia, we investigated if a canonical downstream signaling component of TNFa, nuclear factor kappa-light-chain-enhancer of activated B cells (NFkB) [33], was expressed in glia along DRG sensory afferent axons. We first scored the expression of GFP in *Tg(NFkB*:*egfp);Tg(sox10*:*mrfp)* larvae, which express GFP under control of *nf-kb* binding domains, reliably reporting NFkB activity [34], from 48 to 72 hpf. In these embryos between 48 and 56 hpf, we detected GFP expression in glial cells ensheathing the DRG central projection. Importantly, GFP expression between 48 and 72 hpf was only detected in DRG that had formed a central projection that extended into the spinal cord (48 hpf- 7.5%, 72 hpf-87.5%, n = 30 DRG) (Fig 5A and 5B). By 72 hpf, 87.5% of DRG examined along the length of the zebrafish trunk had GFP^+^ glial cells, suggesting robust activation of NFkB was correlated with the entry of the pioneer axon into the spinal cord (Fig 5B). To confirm this hypothesis, we used time-lapse imaging in *Tg(NFkB*:*egfp);Tg(sox10*:*mrfp)* embryos from 48 to 72 hpf, the period when pioneer DRG axons navigate into the spinal cord. With *Tg(sox10*:*mrfp)*, we could visualize the entry of the growth cone into the spinal cord. In these movies, we did not detect GFP in pioneer axon-associated glia when the central axon formed. However, we observed GFP expression in associated glia soon after the centrally-projecting axon entered the spinal cord and by 72 hpf, GFP was expressed in nearly all pioneer axon-associated glia (Fig 5A and 5B). Based on this data, we conclude that NFkB is active in DRG glia after pioneer axons have entered the spinal cord.

To test if *Tg(NFkB*:*gfp)* expression in DREZ glia is dependent on the pioneer axon entering the spinal cord, we perturbed the navigation of pioneer axons into the CNS by transecting pioneer axons after they formed, but before they successfully navigated to the DREZ in *Tg(NFkB*:*gfp);Tg(sox10*:*mrfp)* embryos [28]. At 72 hpf, 24 hpa, we did not detect GFP in afferent axon-associated glia when pioneer axons were successfully axotomized before they entered the spinal cord (25% GFP^+^ n = 8 DRG) (Fig 5C). To confirm that this lack of expression was not a response to axotomy, we axotomized *sox10*^+^ axons that had already entered the spinal cord at 48 hpf. At 24 hpa, we did not visualize an elimination or reduction of GFP expression in these DRG glia (87.5% GFP^+^ n = 8 DRG) (Fig 5C). As a control, we also axotomized the DRG peripheral projection between 48 and 56 hpf, before afferent projections had navigated into the spinal cord, and then scored GFP expression at 72 hpf. In these studies, we always saw GFP^+^ glia along the afferent projection (Fig 5C). Therefore, these data are consistent with the hypothesis that axon entry is required for NFkB activation in DRG glia.

Because the upregulation of TNFa in DRG neurons correlated with the entry of the pioneer axon into the spinal cord, we sought to understand the temporal dynamics of TNFa upregulation in DRG neurons compared to NFkB activation in associated glia. To do this, we compared intensity profiles from our *Tg(tnfa*:*gfp)* and *Tg(NFkB*:*egfp)* time-lapse movies and normalized the time for the point of axon entry into the spinal cord. These data showed that *Tg(NFkB*:*egfp)* intensity increased at similar time points compared to the time of axon entry (Figs 3D and 5D). Based on this analysis, we conclude that NFkB is upregulated in pioneer axon-associated glia after the pioneer axon enters the spinal cord.

If this model is correct and TNFa-mediated signaling is active in sensory glia as the pioneer axon enters the spinal cord, then perturbation of TNFa should disrupt NFkB expression in associated glia. To test this hypothesis, we injected a *tnfa* MO into *Tg(NFkB*:*egfp)* zebrafish embryos and assayed GFP expression in DRG glia at 72 hpf (Fig 5E and 5F). Consistent with our hypothesis, GFP expression in pioneer axon-associated glia was completely abolished in *tnfa* morphants (*tnfa* MO = 0% GFP^+^ DRG, n = 30 DRG) (Fig 5E and 5F). Based on these data, we conclude that upregulation of TNFa in DRG neurons induces TNFa-mediated expression of NFkB in associated glia.

To dissect this pathway further, we sought to determine if TNFa acted as a cleaved soluble peptide or in its transmembrane form. To test the requirement of TNFa cleavage as a function of glial ensheathment of the DRG central projection, we treated *Tg(sox10*:*eos)* zebrafish embryos with TNFa Protease Inhibitor-1 (TAPI-1), an inhibitor of the metalloprotease *adam17*, which is required for TNFa cleavage [35]. In larvae treated with TAPI-1 from 24 to 72 hpf, we observed that glial ensheathment of DRG pioneer axons was identical to DMSO-treated larvae (S5 Fig). As confirmation that TAPI-1 disrupted metalloprotease activity in our paradigm, we visualized that differentiation of peripheral glia was expedited along the mixed portion of spinal peripheral nerves, a phenotype that is consistent with the lack of MMP activity that has previously been reported (S5 Fig) [36]. From these data, we conclude that TNFa cleavage is not required for proper ensheathment of DRG central axons.

### TNFR2 is required for TNFa-mediated pioneer axon glial ensheathment

TNFa-mediated signaling is modulated through two receptors, tumor necrosis factor receptor 1 (TNFR1) and TNFR2. Previous studies demonstrate that TNFR2, rather than TNFR1, is more likely to signal via transmembrane TNFa [33]. TNFR2 is also exclusively expressed in non-neuronal cells of the DRG [37]. To confirm that *tnfr2* is expressed in glia in the DRG, we designed multiplex fluorescent *in situ* hybridization probes specific to *tnfr2*. We performed *in situ* hybridization analysis with these probes in *Tg(ngn1*:*gfp)* zebrafish embryos and observed that *tnfr2* mRNA was localized around GFP^+^ sensory neurons, consistent with the expression of *tnfr2* in DRG glia at 72 hpf (S1 Fig). We extended this analysis by visualizing both *tnfa* and *tnfr2* expression in *Tg(ngn1*:*gfp)* animals and saw that *tnfa* was exclusively expressed in GFP^+^ sensory neurons while *tnfr2* was expressed in cells surrounding GFP^+^*/tnfa*^+^ sensory neurons (S1 Fig). This data led us to conclude that *tnfr2* is expressed in pioneer axon-associated glia.

Therefore, we investigated the role of TNFa-TNFR2 mediated signaling in pioneer axon glia ensheathment. We first tested if NFkB upregulation in associated glia was dependent on TNFR2. To investigate this, we injected a *tnfr2* MO [29] into *Tg(NFkB*:*egfp)* zebrafish and observed that GFP expression was absent in DRG glia at 72 hpf (*tnfr2* MO = 0% GFP^+^ DRG, n = 30 DRG) (Fig 5E and 5F). These data are consistent with the model that NFkB activity is driven by TNFR2 signaling.

**Fig 6:**
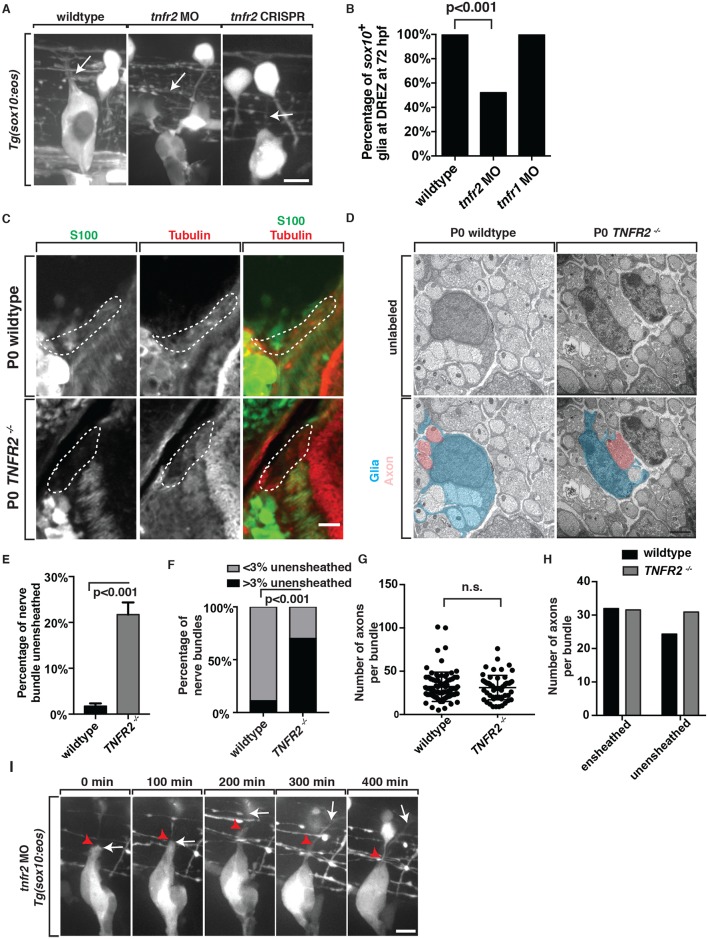
TNFR2 is required for glial ensheathment of DRG axons at the DREZ. (A) Images of *Tg(sox10*:*eos)* wildtype, *tnfr2* morphant and *tnfr2* F0 CRISPR mutant larvae at 72 hpf showing a central branch that is ensheathed by *sox10*^+^ glia (arrow) in wildtype but not *tnfr2* morphants or *tnfr2* F0 CRISPR mutants (arrow). (B) Quantification of data from panel A (wt n = 40 DRG, *tnfr2* MO n = 31 DRG, *tnfr1* = 24 DRG). (C) Images of spinal column tissue from wildtype and *TNFR2*^*-/-*^ P0 mouse embryos labeled with antibodies to S100 (green) and βIII Tubulin (red). Traced area denotes central projection that extends into the spinal cord. (D) Electron microscopy images of DRG tissue from wildtype and *TNFR2*^*-/-*^ pups showed a reduction in the ensheathment of axonal bundles. (E,F) Quantification of ensheathed vs unensheathed bundles in wildtype and *TNFR2*^*-/-*^ pups (3 animals, wt n = 108 nerve bundles, *TNFR2-/-* n = 68 nerve bundles). (G,H) Quantification of the number of axons per small-caliber bundle in wildtype and *TNFR2*^*-/-*^ pups. (I) Images from a 24-hour time-lapse movie of a *Tg(sox10*:*eos)* embryo injected with *tnfr2* MO. Fisher’s exact test (B,F,H). Students t-test (E,F). Scale bars, 25 μm (A,C,I), 5 μm (D).

We next investigated the role of TNFR2 in glial ensheathment of DRG afferent axons. To do this, we injected a translation blocking MO to *tnfr2* into single-cell *Tg(ngn1*:*egfp)* and *Tg(sox10*:*eos)* embryos [29]. Consistent with the hypothesis that TNFa-TNFR2 signaling is required for glial ensheathment of the central projection, we observed a significant reduction in the number of pioneer axons that had associated *sox10*^+^ glia at 72 hpf, which phenocopied *tnfa* morphants (52% contained *sox10*^+^ glia, n = 31 DRG) and was significantly different from wildtype controls (100% contained *sox10*^+^ glia, n = 40 DRG) (Fig 6A and 6B). To confirm that this phenotype was not due to a developmental delay, we scored the ensheathment phenotype at 96 hpf and again visualized defective ensheathment in *tnfr2* morphant larvae when compared to wildtype controls (45% contained *sox10*^+^ glia, n = 44 DRG). To confirm these MO-injected phenotypes were specific to perturbation of *tnfr2*, we injected *Tg(sox10*:*eos)* embryos with gRNAs specific to *tnfr2* and scored glial ensheathment at 72 hpf [30,31]. In these F0 gRNA-injected animals, DRG pioneer axons lacked *sox10*^+^ glial cells. (wildtype 99% contained *sox10*^+^ glia, n = 160 DRG, *tnfr2* 60% contained *sox10*^+^ glia, n = 170 DRG, p<0.0001 students t-test) (Figs 6A and S3C). We confirmed that these injections induced mutations that disrupted the genomic region of *tnfr2* with a T7 endonuclease assay that detected 10 out of the 13 selected larvae were potential mutants (S3B Fig) [30,31]. We then sequenced the genomic region of *tnfr2* in a subset of these individual fish that were scored for defective glial ensheathment to confirm the gRNA induced genomic aberrations at the PAM site (S3E Fig) [30,31]. We also similarly tested the requirement of *tnfr1* in pioneer axon glial ensheathment but did not visualize a phenotype (100% contained *sox10*^+^ glia, n = 24 DRG) (Fig 6B). Based on these data, we propose that *tnfr2* is required for glial ensheathment of DRG afferent axons.

As we did with TNFa, we next asked if TNFR2 played a role in glial ensheathment in mice. To do this, we collected trunk tissue from P0 wildtype and *Tnfrsf1b*^-/-^ (*TNFR2*^*-/-*^) P0 pups, sectioned through the L3-L6 spinal column, and stained the tissue for the glial marker S100 and neuronal marker βIII Tubulin. In wildtype pups, S100 staining labeled cells that associated with Tubulin^+^ axons along the central projection (Fig 6C). However, this labeling was absent or reduced in *TNFR2*^*-/-*^ pups (Fig 6C). Similar to *TNFa*^*-/-*^ pups, *TNFR2*^*-/-*^ pups also had reduced staining of ErbB3 along Isl^+^ sensory axons (S4 Fig). We also quantified this ensheathment phenotype via electron microscopy. In *TNFR2*^*-/-*^ pups, we observed a significant lack of glial ensheathment and organization in the afferent nerve and on average, 21.7% of individual axon bundles were unsheathed, compared to less than 2% of disrupted ensheathment observed in wildtype pups (Fig 6D and 6E, n = 3 animals). Overall the percentage of axonal bundles that were unensheathed was significantly increased in *TNFR2*^*-/-*^ pups when compared to wildtype (Fig 6E and 6F, n = 3 animals) (p<0.0001). In addition, we quantified the number of axons in each small caliber bundle. In wildtype pups, there were on average, 31.5 axons per small caliber bundle, whereas is *TNFR2*^*-/-*^ pups, there 30.9 axons (Fig 6G). When binning the axons bundles into ones that were ensheathed vs unensheathed, the *TNFR2*^*-/-*^ pups had on average, 24.3 that were ensheathed compared to 30.9 that were unensheathed (Fig 6H, n = 3 animals). This is compared to wildtype that had 32.0 ensheathed and 24.3 unensheathed (Fig 6H, n = 3 animals). Based on these data, we propose TNFa-TNFR2 signaling is required for glial ensheathment of the afferent DRG nerve and that TNFa also has a non-TNFR2 function in nerve development (S6C Fig) [26].

### TNFa and TNFR2 within the DRG are required for glial ensheathment

Expression analyses of *tnfa* and *tnfr2* support the hypothesis that TNFa and TNFR2 function within DRG to drive glial ensheathment. To directly test this model, we created mosaic zebrafish larvae by injecting *tnfa* or *tnfr2* MOs into a single cell of 16-cell *Tg(sox10*:*eos)* or *Tg(ngn1*:*gfp)* embryos with a rhodamine-dextran tracer, and then raised those embryos to 72 hpf, when we analyzed glial ensheathment along pioneer axons and scored the cells that contained the rhodamine-dextran marker, and therefore, were MO^+^. In these larvae, we observed normal glial ensheathment of pioneer axons in all larvae that did not have rhodamine-dextran^+^ DRG cells (*tnfa* = 510 nerves, *tnfr2* = 296 nerves) (S3 Table). In contrast, we did observe significant glial ensheathment defects along pioneer axons when cells within the DRG were either rhodamine-dextran^+^/*tnfa* MO^+^ or rhodamine-dextran^+^/*tnfr2* MO^+^ (*tnfa* = 33 DRG, *tnfr2* = 12 DRG) (S3 Table). From these data, we conclude that *tnfa* and *tnfr2* function autonomously within DRG neurons and glia to mediate pioneer axons glial ensheathment.

To gain a deeper understanding of the role of TNFa/TNFR2 signaling in glial ensheathment, we used time-lapse imaging between 48 to 72 hpf in *Tg(sox10*:*eos)* embryos that were injected with a *tnfr2* MO (Fig 6I). In these morphants, we observed that while DRG glia still associated with the single pioneer growth cone as it entered the spinal cord, the glia collapsed back towards the neuronal cell body soon after spinal cord entry, resulting in a naked pioneer axon (Fig 6I). In these movies, we also observed ectopic axonal projections from the DRG in a subset of larvae. Therefore, we scored ectopic projections at 72 hpf in *Tg(ngn1*:*egfp)* larvae and visualized that 44% of *tnfa* morphants and 33% of *tnfr2* morphants had ectopic DRG projections (0% of wildtype animals contained these projections) (S6A and S6B Fig). However, these ectopic axons also lacked glial ensheathment. Based on these data, we conclude that entry of the pioneer axon into the spinal cord induces glial ensheathment via a TNFa/TNFR2-mediated pathway.

## Discussion

The DREZ is a crucial information relay station for the somatosensory circuit [1,2,7]. In this manuscript, we characterize the development of this relay system and identify a signaling cascade that is active during its development. We demonstrate that pioneer axons navigate to the DREZ with supporting glial cells [18] and upon entering the spinal cord, peripheral glia ensheath these axons. We show that this glial ensheathment is dependent on TNFa-TNFR2-mediated signaling. Taken together, our data identify a signaling pathway that is precisely regulated during the entry of DRG pioneer axons into the spinal cord to ensure their ensheathment.

### Axon-glial molecular cross talk

To achieve the precise timing of glial ensheathment during development, axons must coordinate their development with neighboring glia [6,18]. The Neuregulin 1 Type III (Nrg1-III) ligand and ErbB receptors are the most studied example of axon-glial communication that controls the spatiotemporal timing of ensheathment [4,5,19,20]. Nrg1-III is a transmembrane ligand, which until cleaved, ensures only glia in close contact with the axon can bind via ErbB and drive ensheathment and ultimately, myelination [4,19,20]. In this manuscript, we identify another communication mechanism that spatiotemporally controls ensheathment, which requires TNFa and TNFR2. In our model, by controlling the expression of TNFa, ensheathment of the DRG pioneer axon is precisely timed. Spatially, ensheathment is restricted by requiring glia to contact via transmembrane TNFa. It will be interesting to examine in future studies whether axonally-derived *tnfa* is the only source that contributes to *tnfr2*-mediated glial ensheathment. In addition, whether TNFa-TNFR2 signaling also has a role, like NrgI-III/ErbB3, in myelination of these ensheathed axons, remains to be seen, but is an intriguing next step in these studies given that vast literature on NFkB in glial development [13–15,38].

Another interesting and unanswered question of both of these pathways is what induces upregulation of the ligand. Given the temporal upregulation of TNFa that we see as the pioneer axon enters the spinal cord, it is possible that Nrg1-III could be similarly upregulated along axons as they enter nascent tissue. This hypothesis is supported by evidence that muscle-derived neurotrophic factors BDNF and GDNF upregulate Nrg1-III in motor axons [39]. Although our understanding of how axons control their ensheathment is limited, the work we present here implicates an additional signaling cascade that functions in this manner [12].

### A conserved TNFa-mediated signaling pathway

TNFa-mediated signaling is also required for the differentiation of DRG neurons in late stages of embryogenesis [26]. In the absence of TNFa, excess DRG neurons are present in the ganglia of P14 mice, a phenotype that is likely instigated from the failure of those neurons to undergo normal programmed cell death [26]. This neuronal pathway is mediated through TNFR1 and is likely to function when pioneer DRG axons have already entered the spinal cord. In our analysis, TNFa is also required for proper glial ensheathment, but functions through TNFR2. Our results are consistent with the hypothesis that TNFa signaling functions multiple times within development of the sensory circuit, but perhaps distinctly through TNFR1 and TNFR2. For example, we scored an excess of axons in small caliber bundles in *TNFa*^*-/-*^ but not in *TNFR2*^*-/-*^ pups. One potential explanation for this is that TNFa functions in both axonal and glial development whereas TNFR2 has a role only in glial ensheathment, and this may explain the sensory defects previously described in *TNFa*^*-/-*^ pups [26].

Complicating this hypothesis, however, is evidence that DREZ-associated glia are progenitors for DRG cells, which may be defective based on our observation that glial ensheathment is defective. Our data does not distinguish the core cause of our phenotype as loss of glial ensheathment could cause neuronal phenotypes or a phenotype in neurons could drive glial ensheathment defects. Either way, these results introduce a paradigm where TNFa could be used in two distinct pathways with two different cellular outcomes, glial versus neuronal. The developmental timing of these distinct pathways may contribute to their outcomes, shifting TNFa-mediated signaling from a TNFR2 cell arrangement and proliferation cue to a TNFR1-mediated cell death pathway. It is possible that this shift could be controlled by differential expression of the receptors or by variance in cleavage of the TNFa ligand from the membrane. This utility of TNFa during development is further amplified by the fact that the receptors, TNFR1 and TNFR2, can also act as ligands and induce reverse signaling [40]. Identifying candidate downstream components and effectors of NFkB [41], may shed light on this hypothesis.

Although TNFa signaling has been primarily studied in the immune system, it has also recently been shown to be essential for other developmental programs, including hematopoeisis [42]. We believe our discovery that TNFa likely functions as a transmembrane cue to modulate behavior of two contacting cells during neurodevelopment likely has broad implications. For example, this mechanism could be deployed in multiple developmental paradigms such as neuronal migration, cellular tiling or synaptic specificity, where contacting cells must communicate during their development. This possibility significantly expands the potential roles of TNFa during development.

## Materials and methods

### Fish husbandry

All animal studies were approved by the University of Virginia Institutional Animal Care and Use Committee. Zebrafish strains used in this study were: AB*, *Tg(sox10(4*.*9)*:*eos)*^*w9*^ [24], *Tg(sox10*:*mrfp)*^*vu234*^ [43], *Tg(neurod*:*gfp)*^*nl1*^ [23], *Tg(ngn1*:*egfp)*^*w61*^ [23], *Tg(tnfa*:*gfp)*^*pd1028*^ [44] and *Tg(NFkB*:*egfp)*^*nc1*^ [34]. Abbreviations used for each line are denoted in S1 Table. Embryos were produced from pairwise matings and raised at 28.5°C in egg water in constant darkness and staged by hours or days post fertilization (hpf and dpf). Embryos of either sex were used for all experiments [45]. Pigmentation was inhibited for immunohistochemistry and live imaging with phenylthiourea (PTU) (0.004%) in egg water. Stable, germline transgenic lines were used in all experiments.

### Mouse husbandry

All experiments were carried out in compliance with the Association for Assessment of Laboratory Animal Care policies and approved by the University of Virginia Animal Care and Use Committee. Wildtype mice are on a B6;129 mixed background. *TNF*^*-/-*^ and *TNFR2*^*-/-*^ mice were maintained as homozygotes, purchased from The Jackson Laboratory, and were backcrossed to a B6;129 mixed background for four or more generations [26]. Animals of either sex were used for all experiments.

### *In vivo* imaging

PTU-treated embryos were manually dechorionated at 24 hpf and anesthetized with 3-aminobenzoic acid ester (Tricaine), immersed in 0.8% low-melting point agarose and mounted on their right side in glass-bottomed 35 mm Petri dishes (Electron Microscopy Sciences) [46]. A Quorum WaveFX-XI spinning disc confocal system (Quorum Technologies Inc.) equipped with a 25X multi-immersion objective (NA = 0.8), a 40X oil objective (NA = 1.4), a 40X water objective (NA = 1.1) and a 63X water objective (NA = 1.2) mounted on a motorized Zeiss AxioObserver ZI microscope was used to capture images. MetaMorph and Photoshop were used to process images and enhance brightness and contrast of images. Supplementary videos were annotated using ImageJ trackM plugin and formatted using ImageJ.

### Sectioning

Zebrafish embryos and larvae were fixed in 4% PFA for 3 hours at room temperature (25°C) or overnight at 4°C, mounted in sectioning agar and incubated in 30% sucrose overnight at 4°C. For immunohistochemistry, we collected 20 μm transverse sections of the trunk using a cryostat microtome. For mouse tissue, animals were euthanized at P0 and the L3/L6 region of the spinal column was dissected and placed in PFA overnight at 4°C and then 30% sucrose for at least 2 days at 4°C. Tissue was mounted with OCT and then cryosectioned into 20 μm sections.

### Immunohistochemistry

Animals were fixed and stained as previously described [26,46]. The primary antibodies used in this study include: Sox10—1:5,000 [47], Acetylated Tubulin 1:10,000 (Sigma), βIII Tubulin 1:1,000 (Covance), S100 1:1,000 (Dako) [48], ErbB3 1:200 (Santa Cruz) and Isl1 1:100 (Developmental Studies Hybridoma Bank). The secondary antibodies used include Alexa antibodies (Invitrogen) (1:600); goat anti-rabbit 568, goat anti-mouse 568, goat anti-rabbit 647 and goat anti-mouse 647. After staining, zebrafish animals were stored in 50% glycerol/50% 1X PBS until imaged when they were mounted under a bridged coverslip.

### Fluorescent *in situ* hybridization

FISH probes were produced by Biosearch Technologies. Custom Stellaris RNA FISH probes specific to *tnfa* and *tnfr2* probes were made by conjugating 37 different 20 nucleotide oligos specific to *tnfa* mRNA to fluor red 590 dye and by conjugating 40 different 20 nucleotide oligos specific to *tnfr2* to quasar 670 dye. *Tg(ngn1*:*gfp)* zebrafish at 3 dpf were fixed with 4% PFA overnight and then stored in 100% methanol at 4°C. Embryos were mounted and sectioned into 20 μM sections. The BioSearch Technologies protocol for FISH on frozen tissue was used as a guide. Briefly, after sectioning, slides were incubated in PBS for 5 min, then in hybridization buffer (1 ml formamide, 2.5 ml 20X SSC, 10 μl 50 mg/ml Heparin, 500 μl 10 mg/ml tRNA, 10 μl Tween 20, 92 μl 1M Citric Acid, 7.29 ml DEPC Water) for 30 min at 37°C. Probe solution, diluted in 200 μl of hybridization buffer (2 μl of 12.5 μM probe) was then incubated overnight at 37°C. Slides were then washed for 2 hours with PBS, mounted with DAPI slide mount and imaged.

### Eos photoconversion

To image glial dynamics with the pioneer axon, we used *Tg(sox10*:*eos);Tg(ngn1*:*egfp)* embryos treated with PTU, to label DRG precursors with a photoconvertible protein, Eos, that when exposed to UV light, transitions from a green to red emission state, and DRG sensory neurons with GFP [24]. During development, sensory neurons are derived from *sox10*^+^ neural crest cells and then turn on expression of *ngn* when they become post-mitotic neurons [24]. So throughout this imaging, animals were exposed to 450 nm laser for the entire z-stack every 20 minutes to photoconvert the Eos protein. Therefore, in our imaging, sensory neurons were *ngn1*^+^ (green) and also transiently expressed Eos (red) and appeared yellow. All other non-neuronal cells or glia were marked with Eos only and appeared red.

### Pioneer axon axotomy

Axotomy was completed on 48 hpf *Tg(tnfa*:*gfp); Tg(sox10*:*mrfp)* and *Tg(NFkB*:*gfp);Tg(sox10*:*mrfp)* embryos. A ROI was defined and using a high energy pulse with a MicroPoint laser, as previously described, axotomy was performed [28]. After axotomy, a subset of embryos were imaged for 24 hours every 10 min at 40X.

### Morpholino injections

*tnfa*, *tnfr1*, and *tnfr2* MOs [29] were diluted from a stock solution of 1 mM in injection buffer to create a working concentration of 0.5 mM (*tnfa*), 0.6 mM (*tnfr1*) and 0.20 mM (*tnfr2*), as previously described [29]. Each MO was injected into single-cell embryos (S4 Table). Depending on the experiment, embryos/larvae were then mounted or fixed as described above at the appropriate age for the experiment. Morphants that were selected for analysis did not display gross morphological abnormalities, contained normal blood flow, and showed an intact spinal cord with normal peripheral motor nerves exiting the spinal cord within each somite. For quantification of DRG neuronal projections, an abnormal projection was defined as a DRG that had more than one axonal projection that extended to the DREZ. An absence of *sox10*^+^ ensheathment was scored if the afferent nerve contained a thin *sox10*^+^ axonal projection without surrounding *sox10*^+^ glia.

### CRISPR analysis

gRNAs specific to *tnfa* (NM_212859) and *tnfr2* (NM_001089510) were injected in a cocktail with *cas9* RNA (75 ng/ul gRNA, 150 ng/ul cas9) into single cell-embryos [30,31] (S4 Table). At 72 hpf, 16 animals were randomly selected, mounted and imaged. 10 nerves per animal were scored for presence or absence of Sox10^+^ glia at the DREZ. Animals that showed gross morphological phenotypes or spinal cord patterning abnormalities were discarded from analysis. To confirm that gRNAs were generating mutants within *tnfa* or *tnfr2* coding regions, we isolated DNA from 16 randomly selected animals, and performed T7 endonuclease assay as previously described [30,31]. Uninjected animals were used as controls. To confirm that the gRNA was lesioning at the PAM site, we sequenced a subset of these that were imaged to confirm a glial ensheathment defect and compared their sequence to uninjected controls [30,31]. gRNA target sites and PCR genotyping primers are provided in S2 Table.

### TAPI-1 treatment

*Tg(sox10*:*eos)* embryos at 24 hpf were exposed to *TNFa* Protease Inhibitor-1 (TAPI-1) [35] that was diluted 1:1,000 in egg water. At 72 hpf, drug-treated and DMSO-treated control larvae were scored for disruption of DREZ glia and the pioneer axon.

### Fluorescent intensity quantifications

Using MTrackJ plugin of ImageJ, a ROI was identified in the *Tg(sox10*:*mrfp)* channel on the developing DRG neuron and pixel intensity was measured for every time point in the movie. This track was then loaded onto the *Tg(tnfa*:*gfp)* channel and an intensity measurement was similarly identified. The intensity value for GFP and RFP channels at these specific ROIs were determined by ImageJ. These intensity values were then compared to a background ROI in the background black space of the GFP channel. Plotted on the graph is the average of 3 movies for each 5 minute time point in the movie. The values for each time point in each time-lapse movie were calculated by subtracting the background ROI intensity from the ROI intensity of the fluorescent^+^ cell. The ROI used for this measurement can be seen in S3 Movie.

### Mouse electron microscopy

At P0, pups were euthanized and the spinal column with DRG intact was removed by microdissection. Afferent DRG nerves were removed from the spinal cord and DRG and fixed in 4% formaldehyde, processed for electron microscopy and then imaged with an electron microscope. Four nerves from three different animals were processed and imaged per genotype.

### Data quantification and statistical analyses

MetaMorph software was used to generate a composite Z-image for zebrafish cell and nerve counts. Individual z-images were sequentially observed to confirm composite accuracy. All graphically presented data represent the mean of the analyzed data. For mouse quantification, DRG and DREZ from L3-L6 spinal cord nerve regions from 3 individual animals were scored. 20 μm sections, divided into triplicates that could be stained individually for ErbB3 and S100 were quantified. For zebrafish analysis, at least 6 nerves from 5 individual animals were used for quantification. GraphPad Prism software was used to determine statistical analysis. A Fisher’s exact test using a confidence interval of 95% was used in Figs 3G, 4D, 5C and 6B, or a student’s T-test was used in Figs 4F, 4G, 6F and 6G, to determine the level of significance.

## Supporting information

S1 Fig TNFa/TNFR2 signaling components are expressed in the DRG. (A&B) Confocal images of FISH probes specific to *tnfa* and *tnfr2* in *Tg(ngn1*:*gfp)* larvae at 72 hpf showing *tnfa* is expressed in DRG neurons while *tnfr2* is expressed in the DRG consistent with glial expression. Scale bar, 25 μm.
(TIF)

S2 Fig Axotomy of navigating pioneer axon growth cones. Zoomed images of axotomized *Tg(sox10*:*mrfp)* growth cones showing pre-axotomy, 1 hour after axotomy and 24 hours after axotomy. The filopodia-like projections that are typical of a growth cone are absent after axotomy. Note the lack of debris from RFP^+^ glia. Arrows denote growth cone filopodia extensions that are missing after axotomy. Scale bar, 25 μm.
(TIF)

S3 Fig TNFa and TNFR2 F0 CRISPR mutants. (A&B) Gels of T7 endonuclease assay of individual zebrafish embryos injected with gRNA specific to *tnfa* (A) and *tnfr2* (B) showing potential banding patterns, distinct from uninjected animals, that are consistent with a potential mutation. Orange boxes denote digested ban that did not appear in uninjected embryos. (C) Quantification of wildtype, *tnfa* gRNA-injected and *tnfr2* gRNA-injected *Tg(sox10*:*eos)* embryos that display defects in the *sox10*^+^ ensheathment of DRG pioneer axons (wildtype n = 160 DRG, *tnfa* n = 160 DRG, *tnfr2* n = 170 DRG, unpaired t-test). (D&E) Sequences of individual zebrafish embryos injected with *tnfa* gRNA (D) or *tnfr2* gRNA (E) showing specific mutations 3’ of the PAM sequence, validating that these gRNA induce specific mutations within *tnfa* and *tnfr2*. Red letters denote nucleotides that are present in gRNA-injected animals compared to uninjected.
(TIF)

S4 Fig *TNFa*^*-/-*^ and *TNFR2*^*-/-*^ mice have glial defects at the DREZ. (A). Confocal images of wildtype, *TNFa*^*-/-*^ and *TNFR2*^*-/-*^ mouse L3-L6 spinal cord tissue at P0 stained with glial marker ErbB3 and Isl1 showing that glial staining is reduced or absent along the afferent nerve (denoted by orange dotted line). The edge of the spinal cord is labeled with blue dotted line. Scale bar, 30 μm.
(TIF)

S5 Fig TNFa cleavage is not required for glial ensheathment of DRG pioneer axons. Images of DMSO and TAPI-1 treated *Tg(sox10*:*eos)* larvae at 72 hpf. In drug-treated larvae, there is no perturbation to *sox10*^+^ glial ensheathment of central DRG projections. (B) Quantification of glial ensheathment along the peripheral motor nerve showing increased ensheathment in TAPI-1 compared to DMSO. DMSO n = 6 nerves, TAPI n = 12 nerves. Red box denotes the DREZ. Scale bar, 25 μm.
(TIF)

S6 Fig Knockdown of *tnfa* and *tnfr2* causes neural defects. (A) In *Tg(ngn1*:*egfp)* embryos injected with either *tnfa* or *tnfr2* MOs, we observed ectopic axons that did not navigate to the DREZ. (B) Quantification of the number of *ngn1*^+^ cells per DRG at 72 hpf in wildtype, *tnfa* and *tnfr2* morphants. (C) Quantification of *sox10*^+^ glia at 96 hpf in wildtype (n = 60 DRG). The *tnfa* (n = 61 DRG) and *tnfr2* morphant (n = 44 DRG) larvae showing the glial ensheathment defect persists to 96 hpf. Scale bar, 25 μm.
(TIF)

S1 Table Summary of transgenic zebrafish used in this study. (DOCX)

S2 Table List of gRNA targets sites and oligonucleotides used for genotyping. (DOCX)

S3 Table Summary of mosaic analysis of *tnfa* and *tnfr2* knock down on glial ensheathment. Tables showing results from mosaic injection of *tnfa* or *tnfr2* MOs. Left column denotes the number of nerves in a given animal that had disrupted glial ensheathment. Right column denotes the cell-types that were labeled with Rhodamine-dextran in that given animal. Note that Rhodamine-dextran^+^ DRG cells are present in all animals that had glial ensheathment phenotypes. All animals that did not display glial ensheathment phenotypes are listed below the table with cell-types that were labeled with Rhodamine-dextran.
(DOCX)

S4 Table Summary of zebrafish morpholino and gRNA injections. Table shows the gene of interest, concentration of MO or gRNA, number of embryos injected and the number of animals randomly selected for quantifying the number of nerves per animal that were disrupted.
(DOCX)

S1 Movie Pioneer axons navigate directly to the DREZ. Excerpt from a 24-h time-lapse movie of a *Tg(ngn1*:*egfp)* embryo from 48 to 72 hpf. Filopodia-like protrusions extend out of the cell soma and generate a central projection with a growth cone. Eventually, this growth cone crosses into the spinal cord via the DREZ. Frames were captured every 5 minutes and the video runs at 10 fps.
(MOV)

S2 Movie Pioneer axons navigate to DREZ with glia. Excerpt from a 24-h time-lapse movie of a *Tg(ngn1*:*egfp);Tg(sox10*:*eos)* embryo from 48 to 72 hpf that was exposed to UV light to photoconvert Eos every 15 minutes. Note that *sox10*^+^ (red) glia navigate with the growth cone (green) to the DREZ until it enters the spinal cord and then ensheath the axon after entry. Frames were captured every 5 minutes and the video runs at 10 fps.
(MOV)

S3 Movie TNFa is upregulated during spinal cord entry of pioneer axon. Excerpt from a 24-h time-lapse movie of *Tg(tnfa*:*gfp);Tg(sox10*:*mrfp)* embryo from 48 to 72 hpf. After entry of the growth cone (blue square) into the spinal cord, *Tg(tnfa*:*gfp)* intensity increases in the DRG neuron (red and white dot). Left video shows *Tg(tnfa*:*gfp)*, middle shows *Tg(sox10*:*mrfp)* and right shows (merged). Frames were captured every 5 minutes and the video runs at 10 fps.
(MOV)
