# Supplementary figures and images for: TNFa/TNFR2 signaling is required for glial ensheathment at the dorsal root entry zone
Source: PLoS Genet. 2017 Apr 5;13(4):e1006712. doi: 10.1371/journal.pgen.1006712 (PMC5397050; doi:10.1371/journal.pgen.1006712)

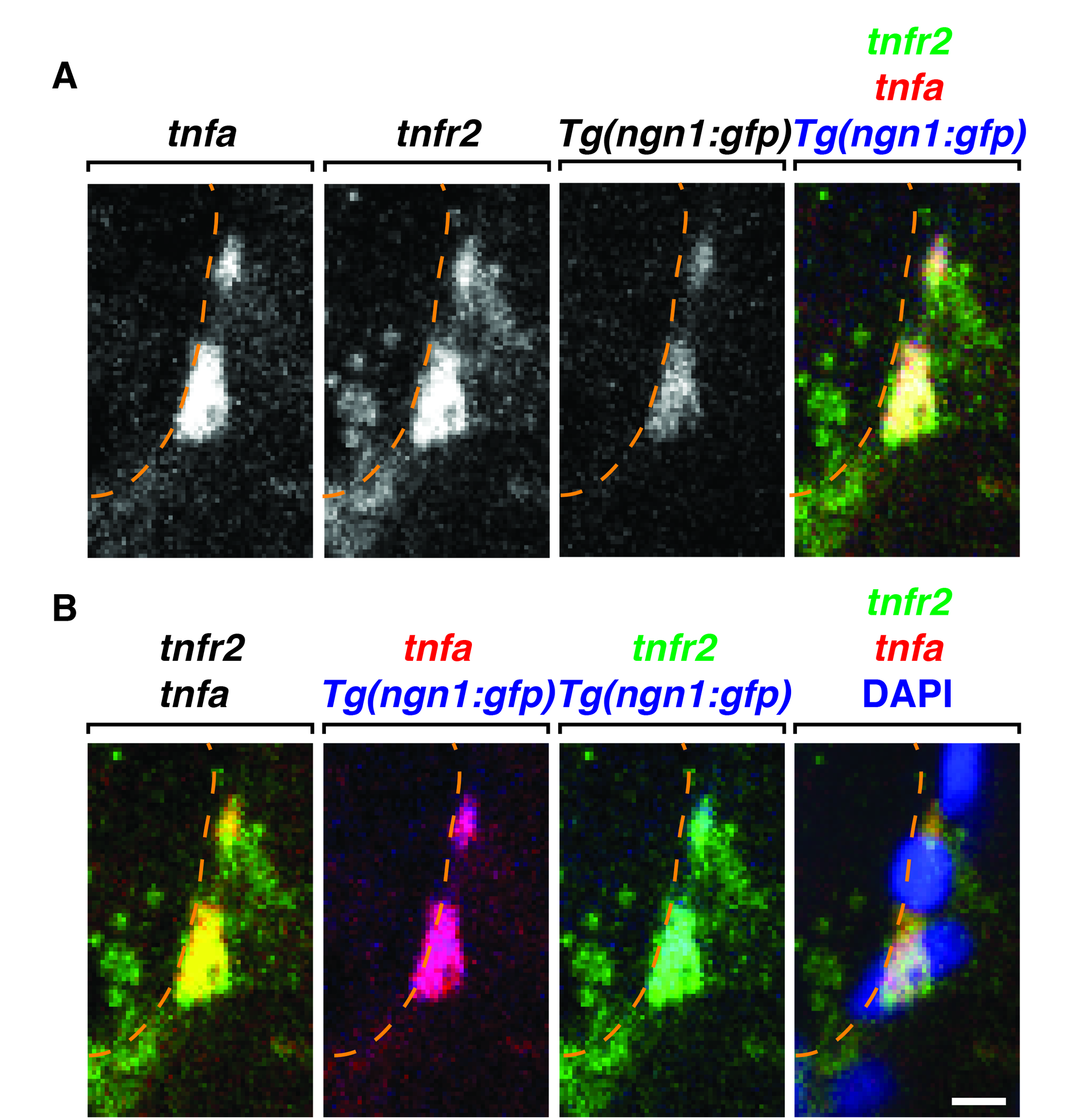

Supplement: S1 Fig — (A&B) Confocal images of FISH probes specific to tnfa and tnfr2 in Tg(ngn1:gfp) larvae at 72 hpf showing tnfa is expressed in DRG neurons while tnfr2 is expressed in the DRG consistent with glial expression. Scale bar, 25 μm. (TIF) [file pgen.1006712.s001.tif]

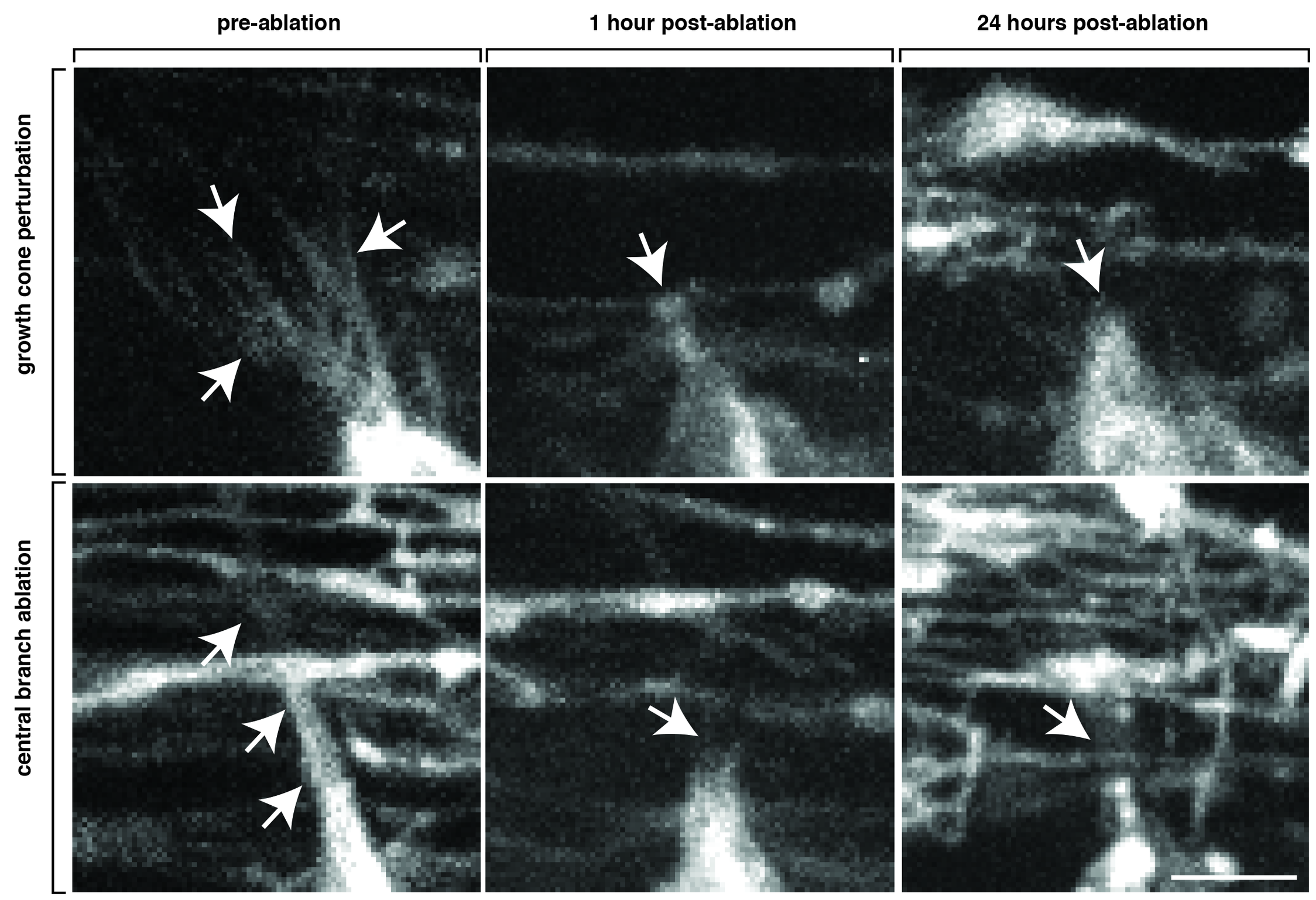

Supplement: S2 Fig — Zoomed images of axotomized Tg(sox10:mrfp) growth cones showing pre-axotomy, 1 hour after axotomy and 24 hours after axotomy. The filopodia-like projections that are typical of a growth cone are absent after axotomy. Note the lack of debris from RFP+ glia. Arrows denote growth cone filopodia extensions that are missing after axotomy. Scale bar, 25 μm. (TIF) [file pgen.1006712.s002.tif]

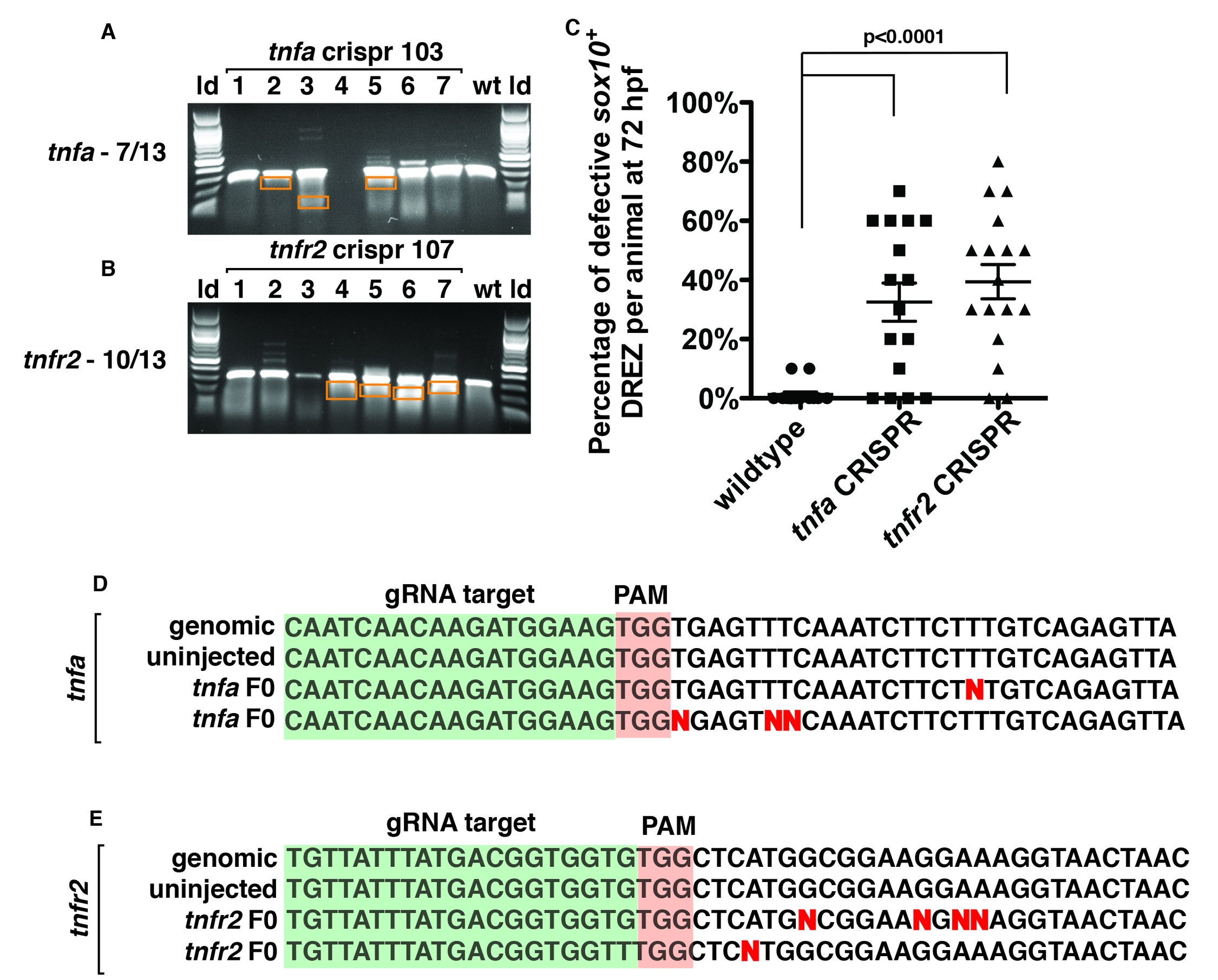

Supplement: S3 Fig — (A&B) Gels of T7 endonuclease assay of individual zebrafish embryos injected with gRNA specific to tnfa (A) and tnfr2 (B) showing potential banding patterns, distinct from uninjected animals, that are consistent with a potential mutation. Orange boxes denote digested ban that did not appear in uninjected embryos. (C) Quantification of wildtype, tnfa gRNA-injected and tnfr2 gRNA-injected Tg(sox10:eos) embryos that display defects in the sox10+ ensheathment of DRG pioneer axons (wildtype n = 160 DRG, tnfa n = 160 DRG, tnfr2 n = 170 DRG, unpaired t-test). (D&E) Sequences of individual zebrafish embryos injected with tnfa gRNA (D) or tnfr2 gRNA (E) showing specific mutations 3’ of the PAM sequence, validating that these gRNA induce specific mutations within tnfa and tnfr2. Red letters denote nucleotides that are present in gRNA-injected animals compared to uninjected. (TIF) [file pgen.1006712.s003.tif]

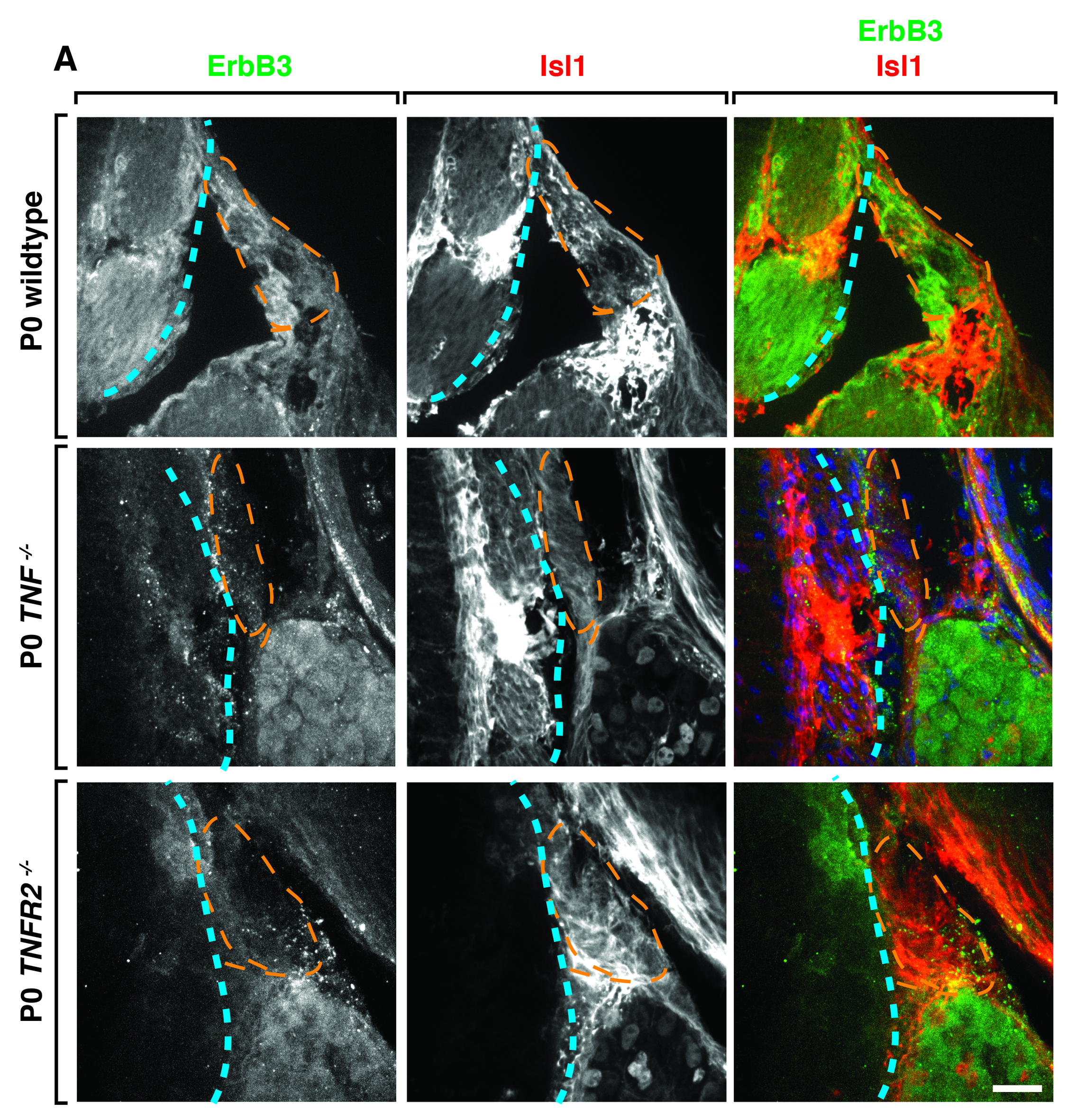

Supplement: S4 Fig — (A). Confocal images of wildtype, TNFa-/- and TNFR2-/- mouse L3-L6 spinal cord tissue at P0 stained with glial marker ErbB3 and Isl1 showing that glial staining is reduced or absent along the afferent nerve (denoted by orange dotted line). The edge of the spinal cord is labeled with blue dotted line. Scale bar, 30 μm. (TIF) [file pgen.1006712.s004.tif]

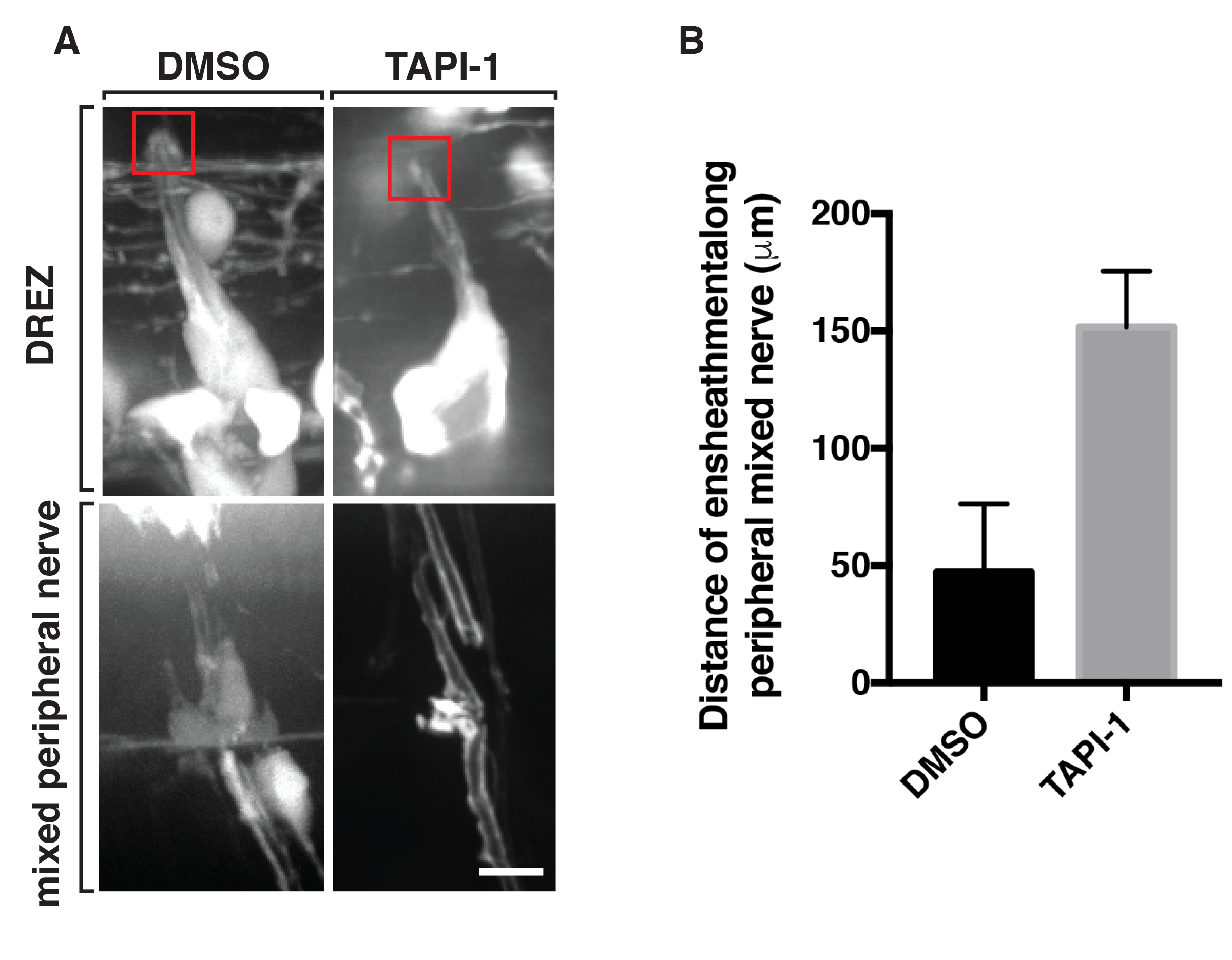

Supplement: S5 Fig — Images of DMSO and TAPI-1 treated Tg(sox10:eos) larvae at 72 hpf. In drug-treated larvae, there is no perturbation to sox10+ glial ensheathment of central DRG projections. (B) Quantification of glial ensheathment along the peripheral motor nerve showing increased ensheathment in TAPI-1 compared to DMSO. DMSO n = 6 nerves, TAPI n = 12 nerves. Red box denotes the DREZ. Scale bar, 25 μm. (TIF) [file pgen.1006712.s005.tif]

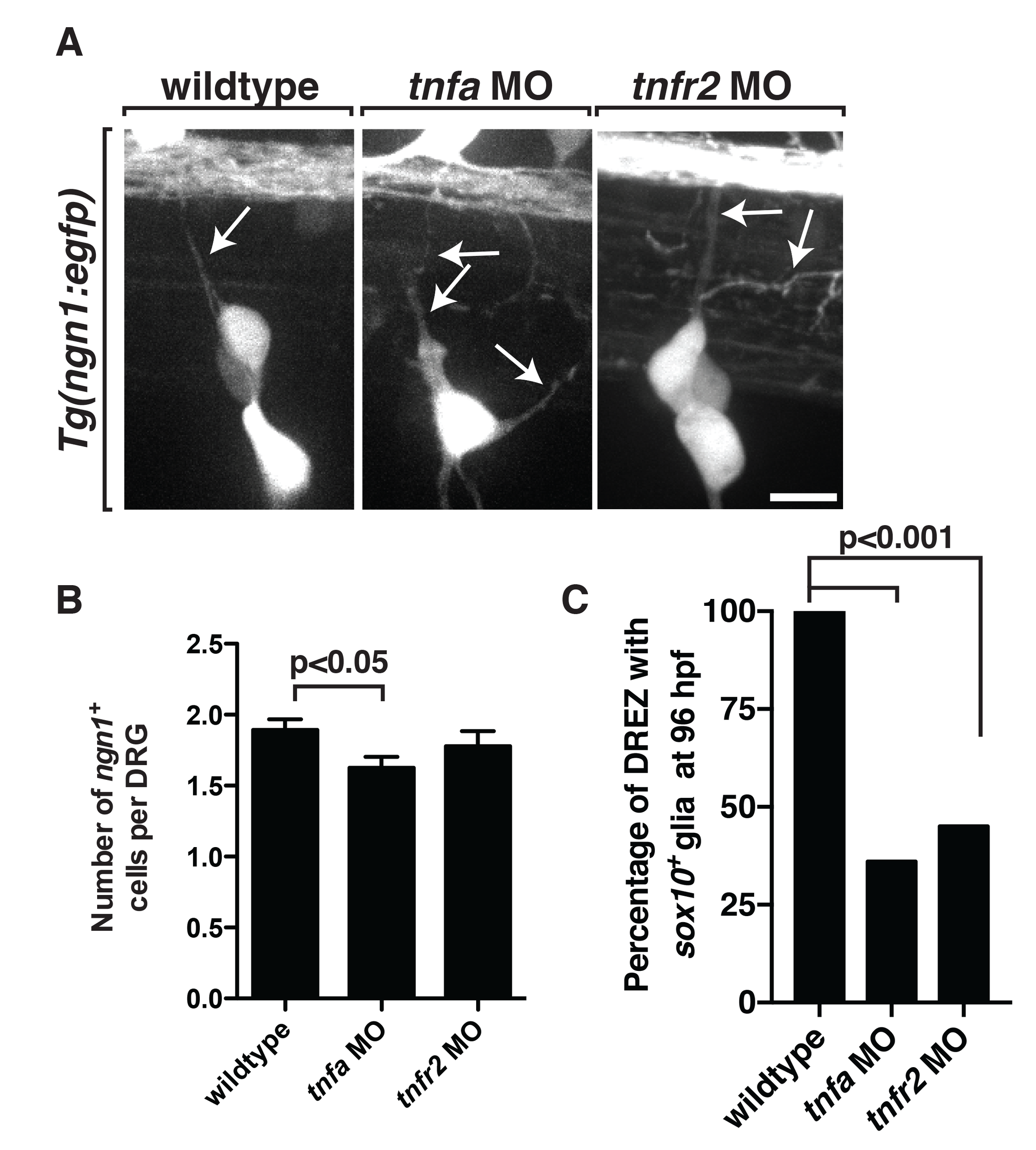

Supplement: S6 Fig — (A) In Tg(ngn1:egfp) embryos injected with either tnfa or tnfr2 MOs, we observed ectopic axons that did not navigate to the DREZ. (B) Quantification of the number of ngn1+ cells per DRG at 72 hpf in wildtype, tnfa and tnfr2 morphants. (C) Quantification of sox10+ glia at 96 hpf in wildtype (n = 60 DRG). The tnfa (n = 61 DRG) and tnfr2 morphant (n = 44 DRG) larvae showing the glial ensheathment defect persists to 96 hpf. Scale bar, 25 μm. (TIF) [file pgen.1006712.s006.tif]
