# Supplementary material for: TNFa/TNFR2 signaling is required for glial ensheathment at the dorsal root entry zone
Source: PLoS Genet. 2017 Apr 5;13(4):e1006712. doi: 10.1371/journal.pgen.1006712 (PMC5397050; doi:10.1371/journal.pgen.1006712)
Supplement: S1 Table — (DOCX) [file pgen.1006712.s007.docx]

| Transgene Name | Transgene abbreviation | Cell Labeled | Transgene action |
| --- | --- | --- | --- |
| *Tg(sox10(4.9):eos)^w9^* | *Tg(sox10:eos)* | DREZ glia, DRG precursors, neural crest | Eos expression in *sox10*^+^ cells |
| *TgBAC(tnfa:GFP)^pd1028^* | *Tg(tnfa:gfp)* | DRG neurons post entry | GFP expression in *tnfa^+^* cells |
| *Tg(neurog1:egfp)^w61^* | *Tg(ngn1:egfp)* | DRG neurons | GFP expression in *ngn1^+^* cells |
| *Tg(sox10(7.2):mRFP)^vu234^* | *Tg(sox10:mrfp)* | DREZ glia, DRG precursors, neural crest | Membrane RFP in *sox10^+^* cells |
| *Tg(NFkB:egfp)^nc1^* | *Tg(NFkB:egfp)* | DREZ glia post axon entry | eGFP expression in NFkB activated cells |
| *Tg(neurod:egfp)^nl1^* | *Tg(neurod:egfp)* | DRG neurons | eGFP expression in *neurod^+^* cells |

Table 1. List of zebrafish transgenes and abbreviations used in text.
