# Supplementary material for: TNFa/TNFR2 signaling is required for glial ensheathment at the dorsal root entry zone
Source: PLoS Genet. 2017 Apr 5;13(4):e1006712. doi: 10.1371/journal.pgen.1006712 (PMC5397050; doi:10.1371/journal.pgen.1006712)
Supplement: S2 Table — (DOCX) [file pgen.1006712.s008.docx]

| gRNA target sequences | | |  |
| --- | --- | --- | --- |
| tnfacrispr 103-125 | cttgagagtcgggcgcttt | Targets exon 1 |  |
| tnfacrispr 142-164 | ttgcctttaccgctggtga | Targets exon 1 |  |
| tnfacrispr | caatcaacaagatggaag | Targets exon 2 |  |
| tnfr2crispr 113-135 | tgttatttatgacggtggtgtgg | Targets exon 1 |  |
| tnfr2crispr 107-129 | attgttgttatttatgacgg | Targets exon 1 |  |
| tnfr2crispr 379-401 | gaaaggactgatgtacggc | Targets exon 4 |  |
| Genotyping primers | |  |  |
| *tnfr2* gen 379 for | GCCTTCAGCATTATAGGCCA |  |  |
| *tnfr2* gen 379 rev | CCGGGTTTGCAGGTTTTGTA |  |  |
| *tnfr2* gen 107 for | AGTGATGGTCAGTGAGTCGC |  |  |
| *tnfr2* gen 107 rev | CAGCGCTGGAGCAAAACATAA |  |  |
| *tnfa* gen 103 for | GACATCACTGGAGTTTCCCCT |  |  |
| *tnfa* gen 103 rev | CCCTCCATACACCCGACTTT |  |  |
| *tnfa* gen old for | AAAGTCGGGTGTATGGAGGG |  |  |
| *tnfa* gen old rev | AATGGATGGCAGCCTTGGAA |  |  |

Table 2. List of gRNA targets sites and oligonucleotides used for genotyping.
