# Supplementary material for: TNFa/TNFR2 signaling is required for glial ensheathment at the dorsal root entry zone
Source: PLoS Genet. 2017 Apr 5;13(4):e1006712. doi: 10.1371/journal.pgen.1006712 (PMC5397050; doi:10.1371/journal.pgen.1006712)
Supplement: S3 Table — Tables showing results from mosaic injection of tnfa or tnfr2 MOs. Left column denotes the number of nerves in a given animal that had disrupted glial ensheathment. Right column denotes the cell-types that were labeled with Rhodamine-dextran in that given animal. Note that Rhodamine-dextran+ DRG cells are present in all animals that had glial ensheathment phenotypes. All animals that did not display glial ensheathment phenotypes are listed below the table with cell-types that were labeled with Rhodamine-dextran. (DOCX) [file pgen.1006712.s009.docx]

***tnfa* Mosaic Animals**

| **Number of nerves disrupted in animal** | **Cell-types labeled** |
| --- | --- |
| 5/10 | **DRG**, spinal cord cells, neural crest derivatives, muscle |
| 2/10 | **DRG**, PNS glia, spinal cord cells |
| 1/10 | **DRG**, spinal cord cells |
| 1/10 | Blood, spinal cord cells |
| 4/10 | **DRG**, spinal cord cells, blood vessels, PNS glia |
| 3/10 | **DRG**, muscle, spinal cord cells |
| 10/10 | All cells |
| 2/10 | Spinal cord cells |
| 5/10 | All cells |

Animals with the following cell-types labeled mosaically did not give a phenotype (51 animals, 510 nerves): muscle, spinal cord cells, pigment cells, blood vessels, radial glia, notochord, intestines, ventral floor plate, interneurons, motor neurons, motor exit point glia, skin, ascending spinal neurons, lateral line neurons, blood cells

***tnfr2* Mosaic Animals**

| **Number of nerves disrupted in animal** | **Cell-types labeled** |
| --- | --- |
| 4/8 | **DRG** |
| 1/8 | **DRG**, muscle, spinal cord cells |
| 2/8 | **DRG**, notochord, spinal cord cells, floorplate |
| 1/8 | **DRG**, spinal cord cells, PNS glia |
| 3/8 | **DRG**, spinal cord cells, PNS glia |
| 1/8 | Spinal cord cells, blood |

Animals with the following cell-types labeled mosaically did not give a phenotype (37 animals, 296 nerves): spinal cord cells, interneurons, blood cells, blood vessels, floor plate cells, radial glia, notochord, muscle, skin, intestines, motor exit point glia, oligodendrocyte progenitor cells, Rohon Beard Neurons, spinal cord neurons

Table 3. Mosaic analysis of *tnfa* and *tnfr2* knock down on glial ensheathment
