# Supplementary material for: TNFa/TNFR2 signaling is required for glial ensheathment at the dorsal root entry zone
Source: PLoS Genet. 2017 Apr 5;13(4):e1006712. doi: 10.1371/journal.pgen.1006712 (PMC5397050; doi:10.1371/journal.pgen.1006712)
Supplement: S4 Table — Table shows the gene of interest, concentration of MO or gRNA, number of embryos injected and the number of animals randomly selected for quantifying the number of nerves per animal that were disrupted. (DOCX) [file pgen.1006712.s010.docx]

**Table S4. Summary of zebrafish morpholino and gRNA injections**

| **Gene Perturbation** | Concentration of MO/gRNA | Embryos Injected | # scored for percentage  showing glial phenotype |
| --- | --- | --- | --- |
| ***tnfa* MO** | 0.5 mM | >100 | 16 |
|  | 1 mM | ~100 | NA* |
| ***tnfr2* MO** | 0.2 mM | >100 | 17 |
|  | 1 mM | ~120 | NA* |
| ***tnfr1* MO** | 0.6 mM | ~100 | 16 |
| ***tnfa* gRNA** | 75 ng/ul | >100 | 16 |
| ***tnfr2* gRNA** | 75 ng/ul | >100 | 16 |

In all experiments, larvae were randomly selected from injected clutches for phenotypic assessment. *These animals showed gross morphological defects in <50% of the injected animals and therefore were not analyzed for percentage of nerves that were defective per animal.
